# P199: Clinical characteristics and therapeutic outcomes of hematogenous vertebral osteomyelitis caused by methicillin-resistant Staphylococcus aureus

**DOI:** 10.1186/2047-2994-2-S1-P199

**Published:** 2013-06-20

**Authors:** MS Lee, KH Park, MH Jung, YS Kim

**Affiliations:** 1 Internal Medicine, Kyung Hee University Hospital, Kyung Hee University School of Medicine, Korea, Republic Of; 2 Infectious Diseases, Asan Medical Center, University of Ulsan College of Medicine, Seoul, Korea, Republic Of

## Introduction

Hematogenous vertebral osteomyelitis (HVO) caused by methicillin-resistant *S. aureus* (MRSA) has increased in recent years. Little information is available regarding the clinical characteristics and outcomes of patients with HVO caused by MRSA, compared with patients with HVO caused by methicillin-susceptible *S. aureus* (MSSA).

## Methods

All patients diagnosed with *S. aureus* (SA) HVO from January 2005 to December 2011 were included in the study. Clinical features and outcomes of MRSA HVO were evaluated compared with MSSA HVO. Molecular and microbiological characteristics of the MRSA isolates were determined.

## Results

Of the 139 patients with SA HVO, MRSA caused 62 (44.6%) cases. Patients infected with MRSA were more frequently of hospital-onset (35.5 vs. 13.0, *P* = .002) than MSSA-infected patients. Based on clinical and microbiological evaluation, a potential portal of entry for SA HVO was identified in 61 patients (43.9%). Intravenous venous catheters were more likely to be the origin in MRSA than in MSSA cases (46.7% vs. 22.6%, *P* = .048). The mortality rates for MRSA and MSSA HVO were similar (21.0% vs. 19.5%; *P* = .83). Longer duration of bacteremia (mean 10.1 vs. 3.1 days; *P* < .001), longer hospital stay (median 69 vs. 52 days; *P* = .001), and more frequent relapse (16.1% vs. 4.3%; *P* = .03) were observed among MRSA cases. Among the MRSA cases, relapse rates were lower in patients with a longer duration of antibiotic therapy: 41.7% (4–6 weeks), 25.0% (6–8 weeks), and 5.6% (≥8 weeks) (*P* = .007). Bacteremia was more likely to persist for ≥7 days in patients with an initial vancomycin trough <15 mg/L than in those with an initial trough ≥15 mg/L (79.3% vs. 20.0%; *P* = .001). A community-associated MRSA strain, specifically ST72-MRSA-SCC*mec*IV, was responsible for 70.8% of community-onset infections and 12.5% of hospital-onset infections.

## Conclusion

MRSA HVO was associated with longer duration of bacteremia, longer hospital stay, and more frequent relapse compared to MSSA HVO. Our data indicate that antibiotic therapy for at least 8 weeks and targeting an initial vancomycin trough of ≥15 mg/L benefit patients with MRSA HVO. Community-associated MRSA strain was responsible for substantial proportion of community-onset MRSA HVO.

## Disclosure of interest

None declared

